# Identification of the hub genes and prognostic indicators of gastric cancer and correlation of indicators with tumor-infiltrating immune cell levels

**DOI:** 10.7150/jca.52105

**Published:** 2021-05-13

**Authors:** Yun Ji, Lu Gao, Can Zhang, Xu Sun, Liping Dai, Zhenyu Ji, Jianying Zhang, Zhida Zhang, Wei Cao, Yang Zhao, Liguo Zhang

**Affiliations:** 1 BGI College, Zhengzhou University, No. 40 Daxue Road, Zhengzhou 450007, China.; 2 Henan Institute of Medical and Pharmaceutical Science, Zhengzhou University, No. 40 Daxue Road, Zhengzhou 450052, China.; 3 Zhengzhou Central Hospital Affiliated to Zhengzhou University, Zhengzhou University, Zhengzhou 450000, China.; 4 Integrated TCM and Western Medicine Department, Affiliated Cancer Hospital of Zhengzhou University, Zhengzhou 450008, China.

**Keywords:** gastric cancer, differentially expressed genes, Hub gene, prognostic indicators, tumor-infiltrating immune cells

## Abstract

**Aims:** To identify the hub genes and prognostic indicators of gastric cancer (GC) and determine the correlation between prognostic indicators and the tumor-infiltrating immune cell levels so as to provide useful information for future GC diagnosis and treatment.

**Methods:** The Cancer Genome Atlas (TCGA) stomach adenocarcinoma dataset and two microarray datasets were used to screen the overlapping differentially expressed genes (DEGs) between normal gastric and GC tissue samples. Hub genes were screened via protein-protein interaction networks and module analysis of the overlapping DEGs. Their expression was validated at the cell level and tissue level using the ONCOMINE database. The prognostic indicators of overall survival (OS) and disease-free survival was identified by Cox proportional hazards regression analysis based on tumor grade and cancer stage. The expression of hub genes was validated at the cell level. The correlation of prognostic indicators with the tumor-infiltrating immune cell levels was analyzed using Tumor IMmune Estimation Resource.

**Results:** Ten hub genes, namely *CDC6*,* CDC20*,* BUB1B*,* TOP2A*,* CDK1*,* AURKA*,* CCNA2*,* CCNB1*,* MAD2L1*, and *KIF11*, were screened and their upregulation in the GC tissue was verified. Three prognostic factors, namely *LUM*,* VCAN*, and *EFNA4*, were identified; their expression was higher in GC cells than in normal cells.* LUM*, *VCAN*, and *EFNA4* were correlated with tumor-infiltrating immune cell levels in GC.

**Significance:** The identified hub genes and prognostic indicators of GC could be useful indicators for future GC diagnosis and treatment.

## Introduction

Gastric cancer (GC) is one of the most common malignant tumors worldwide and the third leading cause of cancer-related mortality after lung and breast cancers 1. The Global Cancer Statistics 2018 report stated that there were over 1,000,000 new GC cases and that approximately 8% of patients with GC died in 2018 2. The high mortality rate associated with GC is owing to its insidious onset, i.e., early symptoms are not obvious. Most patients with GC are diagnosed at the advanced stage; their 5-year overall survival (OS) is only 28.3% 3. However, to date, the aspects ultimately affecting the occurrence, development, and prognosis of GC remain unclear.

Most researchers use bioinformatic methods to study microarray and RNA-sequencing (RNA-seq) data to identify the prognostic indicators and therapeutic targets associated with GC. Cao *et al*. used the Gene Expression Omnibus (GEO) dataset and subsequently identified key diagnostic genes and determined the pathways playing significant roles in GC progression 4. Fei *et al*. identified the important prognostic indicators and pathways for GC treatment using the GEO dataset by identifying overlapping differentially expressed genes (DEGs) 5. However, analysis on the related makers are inadequate and even contradictory owing to their different data processing methods or different sample sizes 6, 7. Nevertheless, a comprehensive bioinformatic method has been applied in the research of various cancers and a large amount of valuable biological information has been discovered; this has made it possible to find useful and reliable molecular markers 8.

In this study, we performed comprehensive bioinformatics analysis to simultaneously analyze two microarray datasets and RNA-seq data of human GC and normal gastric tissue samples so as to identify the hub genes and prognostic indicators of GC. Further, the correlation between the identified novel prognostic indicators and tumor-infiltrating immune cell levels was verified to identify the possible role of these indicators in cancer immunoregulation.

## Methods

### Data and sources

A set of RNA-seq data of GC was downloaded from The Cancer Genome Atlas (TCGA). UCSC Xena database (http://xena.ucsc.edu/) which contains normal stomach tissues and TCGA-STAD tissues was utilized. Another three gene expression arrays of human GC datasets (GSE13911, GSE79973 and GSE56807) 9-11 were obtained from GEO. Further, the clinicopathological information and survival data of 443 patients with GC were obtained from TCGA. For further analysis, considering the factors that are not associated with disease mortality may bias the survival analysis, samples with a survival time of less than 90 days as well as those without gender, age, tumor pathological stage, and corresponding transcriptome data were eliminated. Finally, 317 samples that met the admission criteria were included in this study. Additionally, the disease-free survival (DFS) data of 246 patients with stomach adenocarcinoma (STAD) were acquired from cBioPortal (https://www.cbioportal.org/).

### Identification of DEGs

DEGs were identified by comparing the normalized expression data of GC and adjacent normal tissues using the limma package in R software. DEG intersection was performed in three datasets, and |log2FC| ≥ 1, *P*-value of <0.05, and adjusted *P*-value of <0.05 were considered statistically significant. The method of adjusted *P*-value was Benjamini & Hochberg (BH). The expression of overlapping DEGs was based on that of STAD in TCGA.

### Functional enrichment analysis of overlapping DEGs

To determine the biological functions and potential signaling pathways of overlapping DEGs, Gene Ontology (GO) and Kyoto Encyclopedia of Genes and Genomes (KEGG) pathway enrichment analyses were performed using the clusterProfiler 12 and org.Hs.eg.db 13 packages in R. The cutoff criteria were a *P*-value of <0.01 and an adjusted *P*-value of <0.05.

### Hub genes and module analysis

The protein-protein interaction (PPI) network analysis of overlapping DEGs was performed using the STRING database 14. A confidence score of ≥0.41 was selected to construct the PPI network with overlapping DEGs in Cytoscape version 3.7.1. CytoHubba 15 and Molecular Complex Detection 16 were used to screen the hub genes and perform module analysis, respectively.

### ONCOMINE analysis

The ONCOMINE (www.oncomine.org) database was used to validate the expression of the 10 hub genes at the tissue level. In this study, a *P*-value of 0.05, a fold change of 2, and a gene rank in the top 10% were set as the significance thresholds. The data type was mRNA, and the Student's t-test was used to analyze the differences in the expression of the 10 hub genes in GC.

### Survival analysis and establishment of the prognostic model

The data of 317 patients with GC, including the expression of overlapping DEGs, survival time (>90 days), and survival rate, were analyzed using the R package “survival” (R package version 2.38, https://CRAN.R-project.org/package=survival) in order to perform univariate Cox proportional hazards regression analysis. To identify prognostic indicators, overlapping DEGs with a *P*-value of <0.05 related to survival were selected as candidate genes and used for multiple Cox proportional hazards regression analysis. The following function was used: risk score = expression of gene1 × β1gene1 + expression of gene2 × β2gene2 + … expression of gene(n) × β(n)gene(n) 17. Patients were classified into low-risk and high-risk groups based on their median prognostic risk score. Protective genes [hazard ratio (HR) < 1] or risk genes (HR > 1) were identified by calculating HR and 95% confidence interval (CI). The R package “survivalROC,” which performs time-dependent receiver operating characteristic curve analysis on 5-year OS data, was used to evaluate the performance of the prognostic model. A survival curve was generated using “survival” and “survminer.” The R software (version 3.6.1) was used for all statistical analyses. The survival package in R was used to explore the DFS of single gene signatures as potential prognostic genes. Based on the median expression of target genes, patients were divided into low-expression and high-expression groups.

### UALCAN analysis and Gene Expression Profiling Interactive Analysis (GEPIA2)

The relative expression of three prognostic genes, namely *VCAN*, *EFNA4*, and *LUM*, in different tumor subgroups based on clinicopathological criteria, such as tumor stage and tumor grade, were analyzed using UALCAN 18 (http://ualcan.path.uab.edu/). GEPIA2 was used to study the survival rate of patients based on the expression of 13 prognostic genes, including the three prognostic genes with different isoforms (*VCAN*, *EFNA4*, and *LUM*) 19.

### Analysis of tumor-infiltrating immune cells

Tumor IMmune Estimation Resource (TIMER), an online tool, with Spearman's method, was used to determine the potential correlation between prognostic genes and tumor-infiltrating immune cells, including B cells, CD4^+^ T cells, CD8^+^ T cells, neutrophils, macrophages, and dendritic cells 20 (https://cistrome.shinyapps.io/timer/).

### Cell lines and cell culture

Two GC cell lines (SGC-7901 and MGC-803) and a normal human gastric mucosal cell line (GES-1) were purchased from the Chinese National Infrastructure of Cell Line Resource. The MGC-803 and GES-1 cell lines were cultured in 90% Dulbecco's Modified Eagle's medium (Invitrogen, Carlsbad, USA) supplemented with 10% fetal bovine serum (HyClone, USA). The SGC-7901 line was cultured in 90% RPMI-1640 (Invitrogen, Carlsbad, USA), supplemented with 10% fetal bovine serum (HyClone, USA).

### Real-time quantitative PCR

Total RNA was isolated from whole-cell lysates using the TRIzol reagent (Solarbio, China). cDNAs were synthesized using the PrimeScript^TM^ RT Reagent Kit (TaKaRa, USA) with the gDNA Eraser. Real-time quantitative PCR was performed using TB Green Premix Ex Taq^TM^ II (Tli RNaseH Plus, TaKaRa, USA). GAPDH was used as an endogenous control. The details of the primers are outlined in Additional file 1: Supplementary Table S1.

## Results

### DEG identification from the three datasets

**Figure 1:**
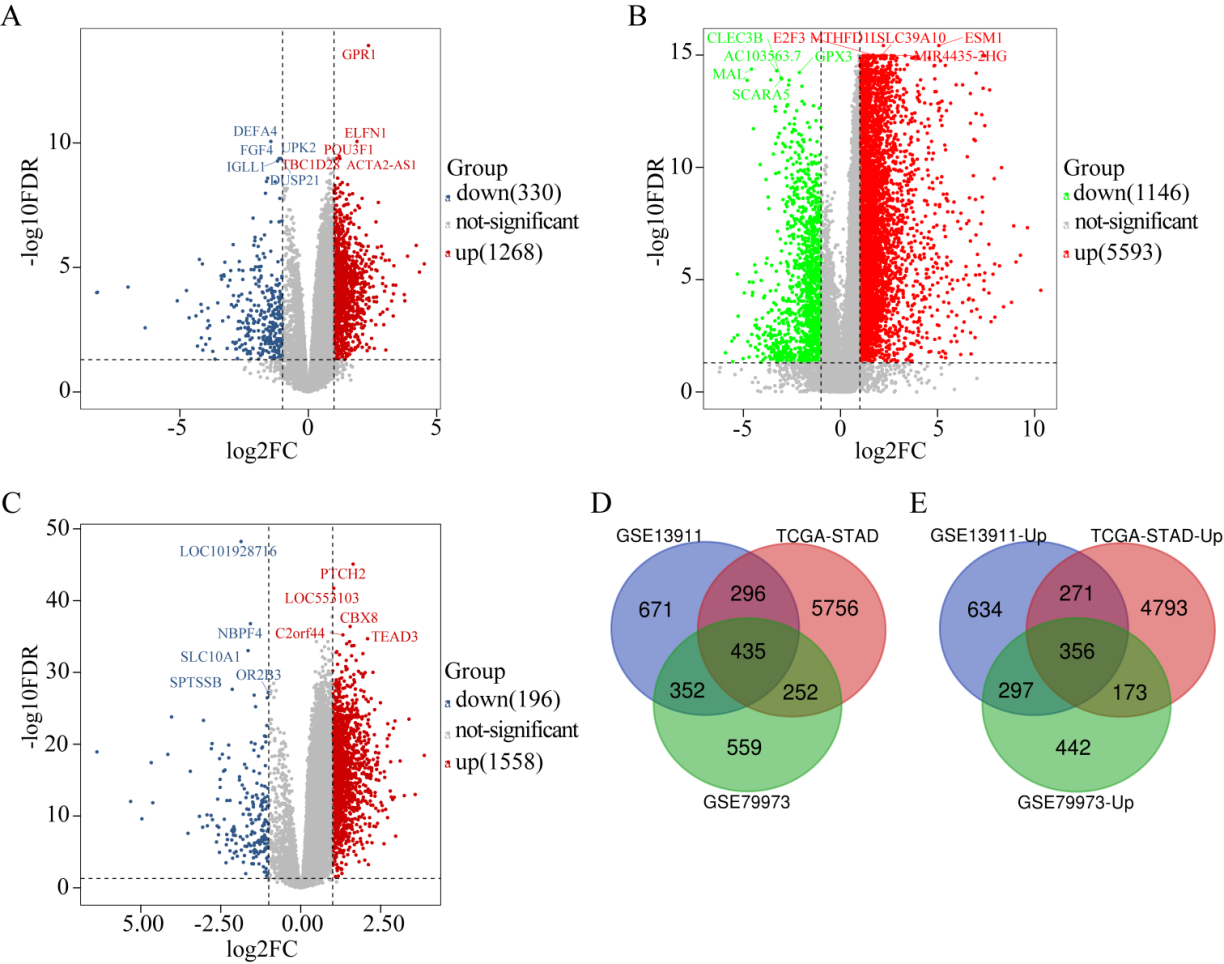
Identification of DEGs in GC (|log2FC| ≥ 1, P < 0.05). (A) The volcano maps of 1268 up-regulated genes (red dots) and 330 down-regulated genes (blue dots) in the microarray dataset GSE79973; (B) The volcano maps of 5593 up-regulated genes (red dots) and 1146 down-regulated genes (green dots) from the TCGA GC dataset; (C) The volcano maps of 1558 up-regulated genes (red dots) and 196 down-regulated genes (blue dots) in the microarray dataset GSE13911; (D) Venn diagrams of the DEGs between the microarray dataset GSE79973, the microarray dataset GSE13911 and the TCGA GC dataset. (E) Venn diagrams of the up-regulated DEGs between the microarray dataset GSE79973, the microarray dataset GSE13911, and the TCGA GC dataset.

The DEGs obtained from GSE79973 included 1268 upregulated genes and 330 downregulated genes (Figure 1A). The DEGs obtained from the TCGA GC dataset included 5593 upregulated genes and 1146 downregulated genes (Figure 1B). Further, the DEGs obtained from GSE13911 included 1558 upregulated genes and 196 downregulated genes (Figure 1C). A total of 435 overlapping DEGs, including 356 upregulated genes (Figure 1E) and 79 downregulated genes, were identified by intersecting the DEGs obtained from the two microarrays and RNA-seq data analysis (Figure 1D).

### Functional enrichment analysis of DEGs

**Figure 2:**
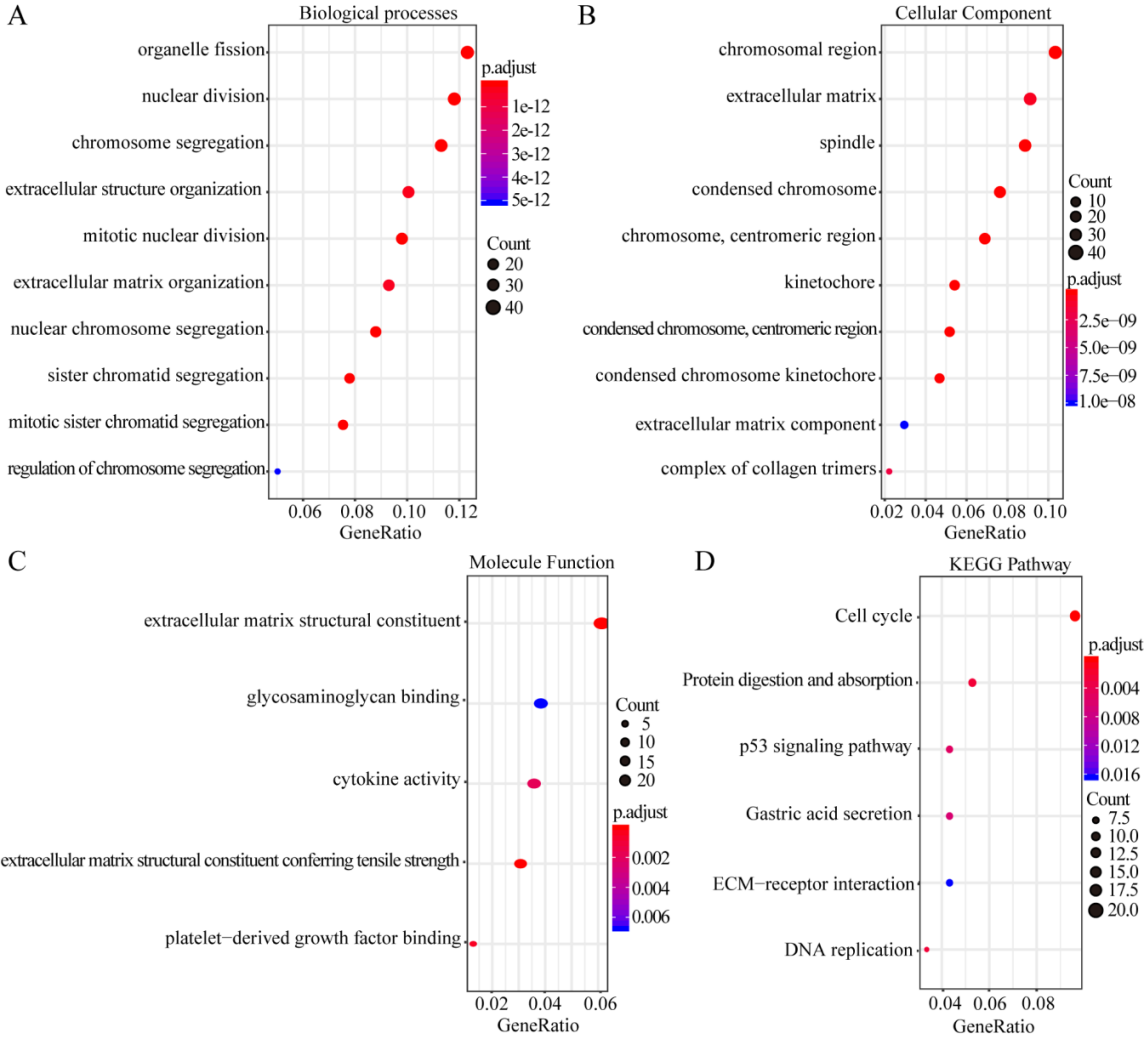
GO enrichment and KEGG pathway analysis of the overlapping DEGs. Biological process GO terms for overlapping DEGs (A), Cellular component GO terms for overlapping DEGs, and (B) Molecular function GO terms for overlapping DEGs (C).The y-axis shows significantly enriched GO terms, and the x-axis shows different gene ratios. The different colors and sizes of the circles represent different *P*-value ranges and contents. The rich factor refers to the ratio of the number of DEGs enriched in a GO term to the number of all the annotated genes enriched in the GO term. In enriched KEGG pathways of DEGs, (D) the y-axis shows enriched pathways, and the x-axis shows different gene ratios; the different colors and sizes of the circles represent different *P*-value ranges and contents. The rich factor refers to the ratio of the number of DEGs enriched in a KEGG pathway to the number of all the annotated genes enriched in the KEGG pathway.

GO enrichment analysis of the biological processes indicated that the overlapping DEGs were mainly enriched in organelle fission, nuclear division, chromosome segregation, extracellular structure organization, and extracellular matrix organization (Figure 2A). Enrichment of cellular component GO terms showed that the 435 overlapping DEGs were mainly enriched in the chromosomal regions, extracellular matrix, and spindle cell components (Figure 2B). In addition, enrichment of molecular function GO terms showed that the main molecular functions of these overlapping DEGs were cytokine activity, glycosaminoglycan binding, and structural component formation in the extracellular matrix (Figure 2C). KEGG pathway analysis revealed that the overlapping DEGs participate in diverse metabolism-associated signaling pathways, including the cell cycle, DNA replication, protein digestion and absorption, p53 signaling, gastric acid secretion, and ECM-receptor interactions. The cell-cycle pathway is the main pathway enriched by the overlapping DEGs (Figure 2D).

### Identification of hub genes and key module

**Figure 3:**
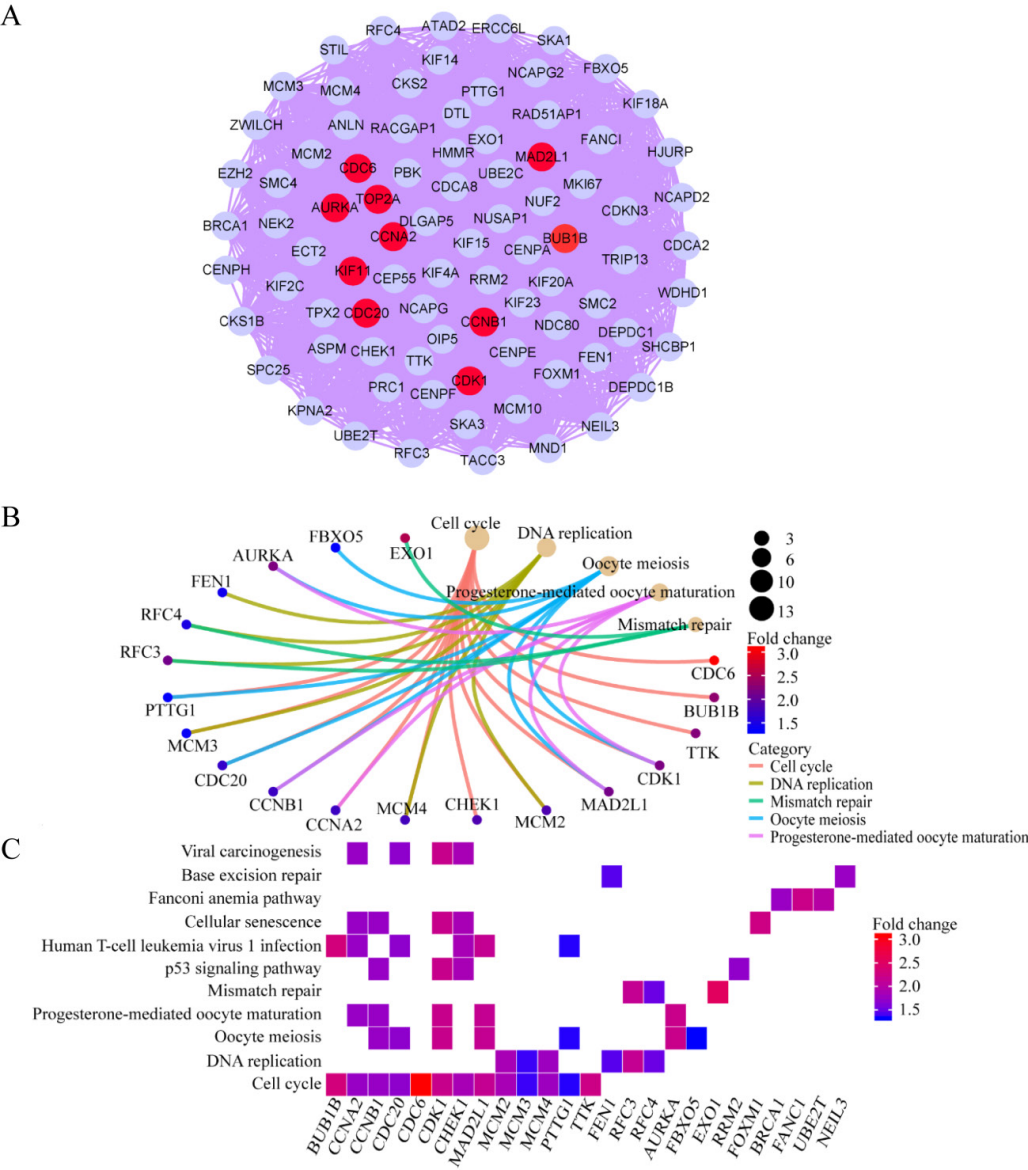
Hub clustering module1and KEGG analysis. (A) Module 1 (MCODE score = 77.095). (B, C) KEGG analysis for genes in Module 1. All circles represent up-regulated genes, and red circles represent hub genes.

The overlapping DEGs were analyzed via PPI network analysis and 380 nodes and 5491 edges were identifieds. Topological feature analysis of the overlapping DEGs led to the identification of 10 hub genes, namely *CCNB1*,* CDK1*,* MAD2L1*,* AURKA*,* BUB1B*,* CCNA2*,* CDC6*,* KIF11*,* TOP2A*, and* CDC20* (Figure 3A). Within this PPI network, 13 modules were obtained via module analysis. The highest scoring module was module 1 (Figure 3A), with a score greater than five times that of the other modules. Additionally, the 10 candidate hub nodes were mainly included in module 1 with all overexpressed genes. This indicates that module 1 represents the key biological characteristics of the PPI network.

GO enrichment analysis revealed that the functions of the genes involved in module 1 are notably enriched in ATPase activity, histone kinase activity, nuclear division, chromosome segregation, the chromosome centromeric region, and spindles. KEGG enrichment analysis revealed that the cell cycle, DNA replication, and oocyte meiosis pathways are the main pathways enriched by the genes in module 1. Among the 10 hub genes, 7 (*CCNB1*,* CDK1*,* MAD2L1*,* BUB1B*,* CCNA2*,* CDC6*, and* CDC20*) are included in module 1 and are enriched in the cell-cycle pathway (Figure 3B and C). This finding further verifies that the hub genes in module 1 could be closely correlated with the cell cycle.

### Verification of the expression levels of the 10 hub genes

**Figure 4:**
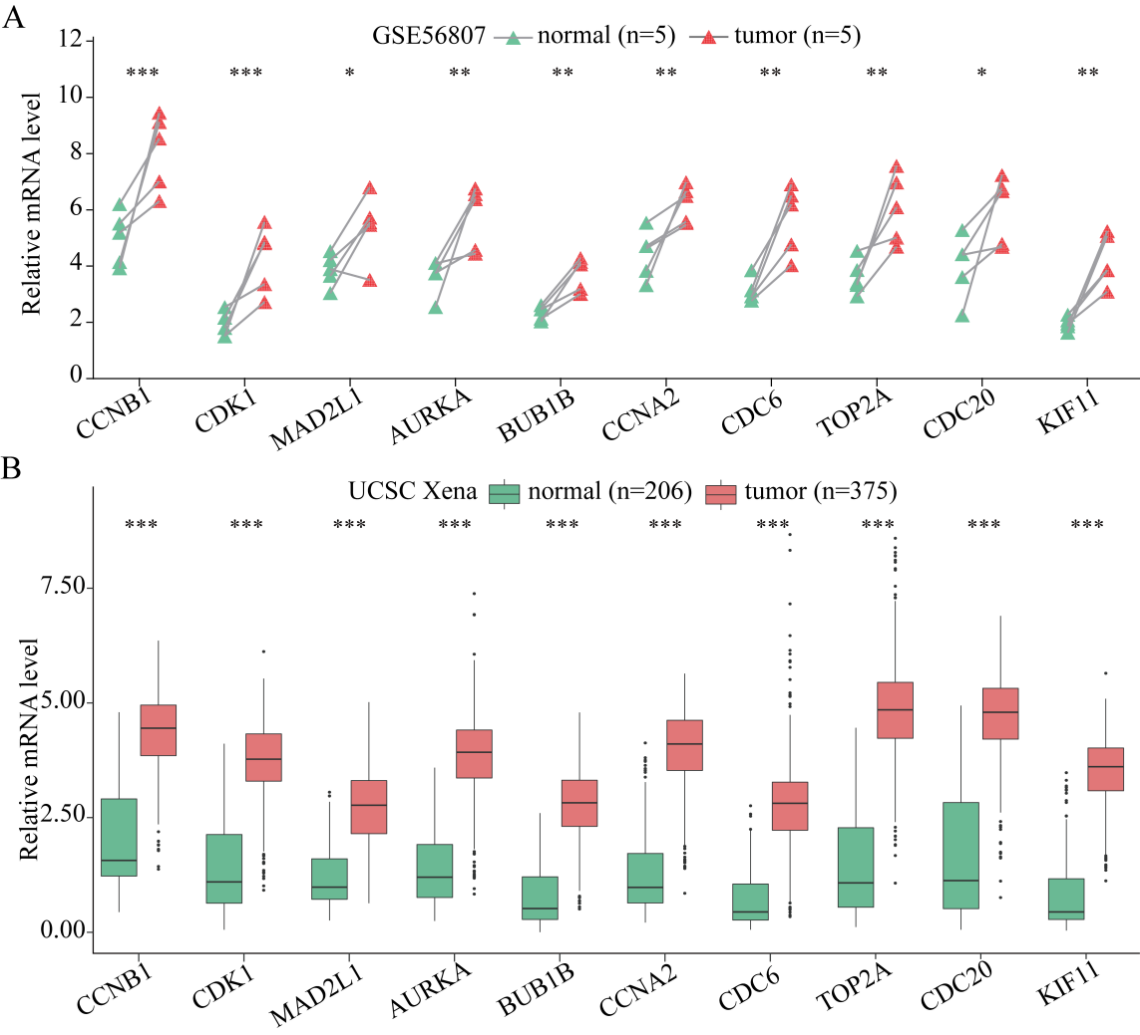
The expression of the 10 hub genes in GC and normal gastric tissues datasets. (A) The expression of the 10 hub genes in GSE56807 dataset with paired 5 GC and 5 normal gastric tissues samples. (B) The expression of the 10 hub genes in the integrated 206 normal gastric tissues samples and 375 GC tissues samples from UCSC Xena database. Expression values of genes are log2-transformed. *P< 0.05; **P < 0.01; ***P < 0.001.

The expression of the 10 hub genes identified for GC was analyzed at the tissue level via using the ONCOMINE database. As shown in Figure S1, these 10 hub genes were shown to be obviously upregulated in GC samples, except for *KIF11*. The expression of *KIF11* in GC tissue is not particularly significant, although its FC (2.04 listed in Table S2) is still more than 2. Therefore, the expression of *KIF11* indicates that it is still upregulated in GC tissue, even though the expression is not significant. Moreover, the expression of these 10 hub genes was verified using GSE56807 dataset and the dataset from UCSC Xena database which is integrated with TCGA and GTEx. As shown in Figure 4, the expression of all the 10 hub genes were demonstrated to be significantly upregulated in GC samples.

**Figure 5:**
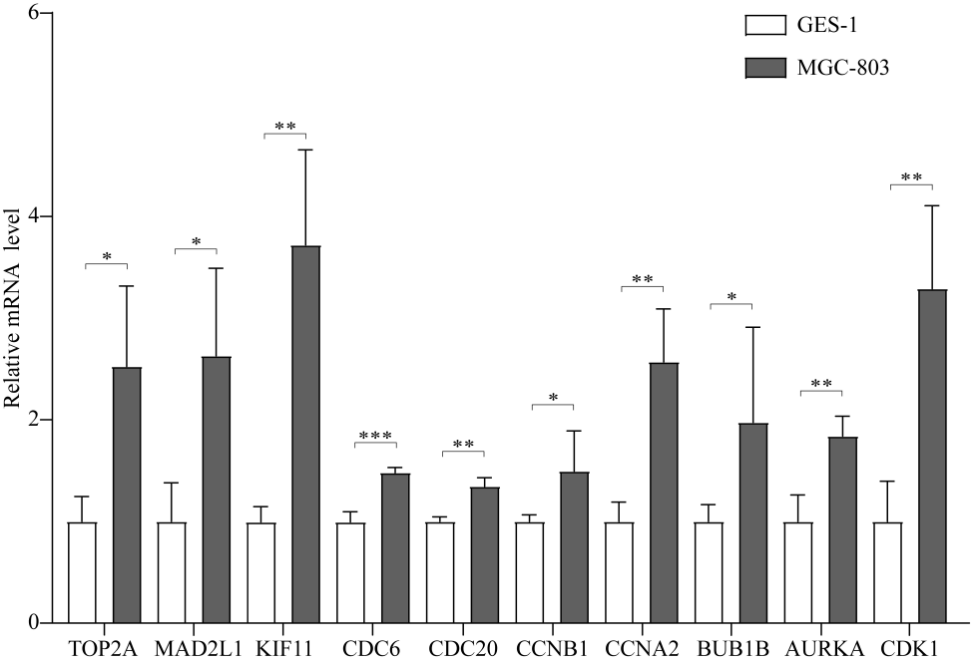
The expression of the 10 hub genes in the GES-1 and MGC-803 cell lines. *P < 0.05; **P < 0.01; ***P < 0.001.

In addition, the expression of these 10 hub genes was verified in MGC-803 and GES-1 cells, as shown in Figure 5. All the 10 hub genes were demonstrated to be significantly upregulated in GC cells compared with that in normal cells. Their expression in GC cells was consistent with that in GC tissues, confirming that these genes are upregulated in GC.

### Identification of prognostic gene signatures

The results of univariate Cox proportional hazard regression model analysis revealed that approximately 42 genes were found to be significantly related to survival time (*P* < 0.05). Multivariate Cox proportional hazard regression model analysis revealed that the prognostic gene signature contained the following 13 genes: *LINC01094*,* CKMT2*,* LUM*,* PSCA*,* TFF1*,* FAP*,* VCAN*,* FEN1*,* CTHRC1*,* CDC6*,* PRRX1*,* EFNA4*, and* PMEPA1* (Table 1).

**Figure 6:**
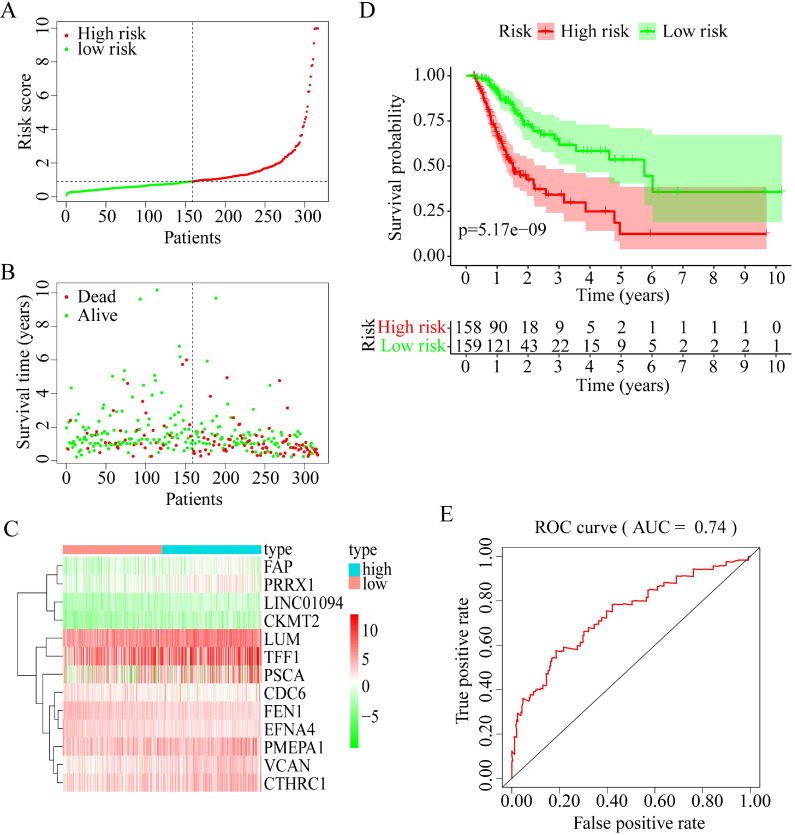
13 prognostic signatures identified from the overlapping 435 DEGs. (A) The risk score distribution. (B) The patients' survival status distribution. (C) The heat map of the 13 genes for low- and high-risk groups. In the heat map, each column represents one sample, and each row represents one gene, and the color gradient ranging from cyan-green to red represents the changing process from down- to upregulation. (D) The Kaplan-Meier curves for low- and high-risk groups. (E) The ROC curves for predicting OS in GC patients by the risk score.

Among these 13 genes, *LUM*,* EFNA4*,* FEN1*, and *FAP* had an HR of <1 and were considered protective prognostic genes, whereas *LINC01094*,* CKMT2*,* PRRX1*,* VCAN*,* CTHRC1*,* CDC6*,* PMEPA1*,* PSCA*, and *TFF1* had an HR of >1 and were considered risk prognostic genes. Figure 6A-C shows the prognostic risk score results. OS was significantly different between the high-risk and low-risk groups (*P* < 0.0001, Figure 6D). In particular, the 5-year OS was 53.52% (95% CI = 40.40-70.90%) in the low-risk group and 12.43% (95% CI = 4.02-38.50%) in the high-risk group. The AUC was 0.74 for 5-year OS, implying that the prognostic gene signature performed well in survival prediction (Figure 6E).

### OS and DFS analyses of the 13 genes

**Figure 7:**
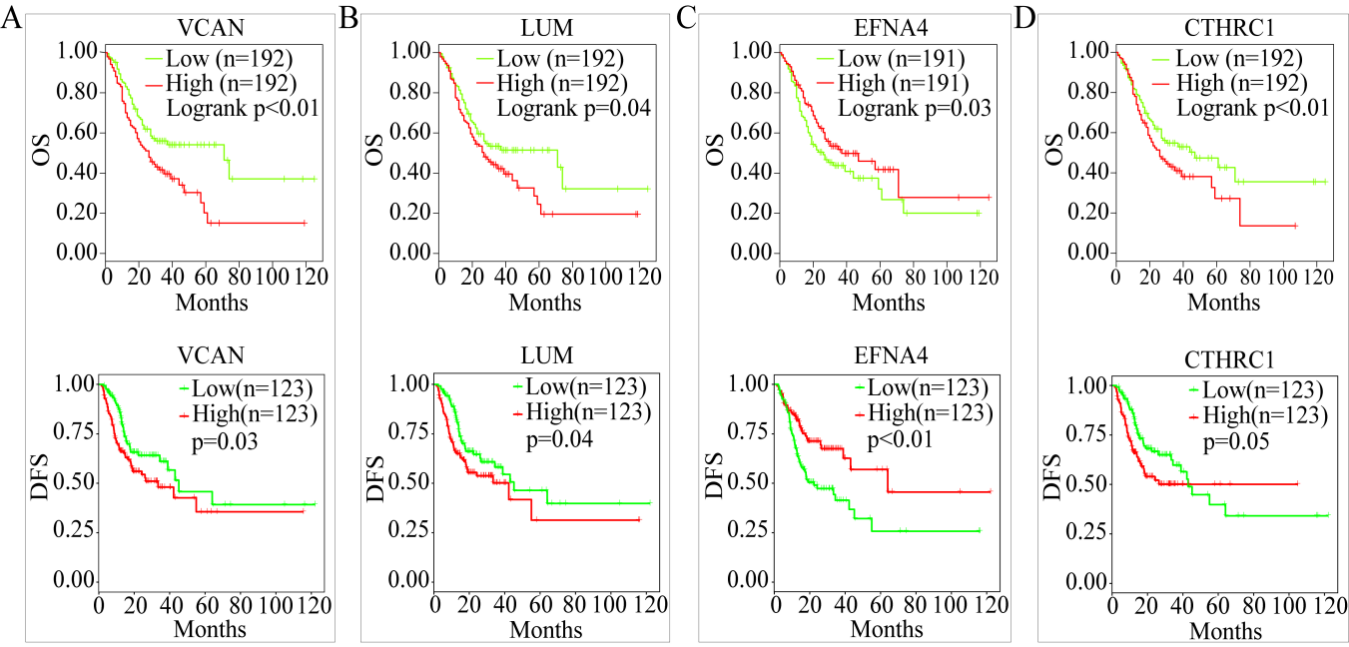
OS and DFS analysis. (A) OS and DSF analysis of *VCAN*. (B) OS and DFS analysis of *LUM*. (C) OS and DFS analysis of *EFNA4*. (D) OS and DFS analysis of *CTHRC1.*

OS analysis of the GC samples in TCGA consistently revealed that the OS with *VCAN*,* LUM*,* EFNA4*, and *CTHRC1* in low-candidate-gene content groups and high-candidate-gene content groups were different (*P* < 0.05) (Figure 7A-D).

However, there was no difference in the OS with *CKMT2*,* FEN1*, *PRRX1*,* LINC01094*,* FAP*,* PMEPA1*,* PSCA*,* TFF1*, and *CDC6* (*P* > 0.05) (Figure S2). Interestingly, DFS analysis of 246 GC samples in TCGA revealed that between the low-candidate-gene content groups and high-candidate-gene content groups, the DFS with *VCAN*, *LUM*, and *EFNA4* was different (*P* < 0.05) (Figure 7A-C). Except for *FEN1*, there was no difference in the DFS with *CKMT2*,* CTHRC1*,* PRRX1*, *LINC01094*, *FAP*, *PMEPA1*, *PSCA*, *TFF1*, and *CDC6* (*P* > 0.05) (Figure S3).

Therefore, *LUM*,* VCAN*, and *EFNA4*, with obvious differences in their OS and DFS, were considered potential prognostic genes.

### Expressions of the potential prognostic genes in the GC subgroup

**Figure 8:**
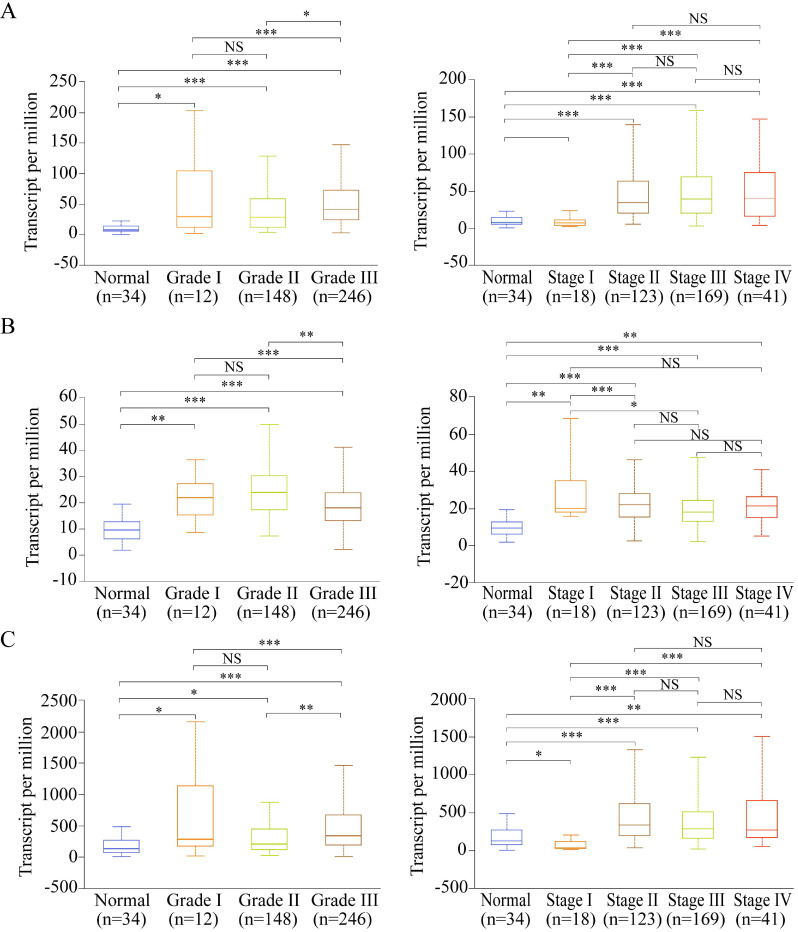
Expression of the potential prognostic genes in GC subgroup. (A) Expression of *VCAN* in STAD based on tumor grade and individual cancer stage. (B) Expression of *EFNA4* in STAD based on tumor grade and individual cancer stage. (C) Expression of *LUM* in STAD based on tumor grade and individual cancer stage. *P<0.05; **P<0.01; ***P<0.001; NS: not significance.

Among the 13 genes, the following three genes had significant differences in both OS and DFS: *LUM*, *EFNA4*, and *VCAN*. Therefore, they were defined as the potential prognostic genes. Based on subgroup analysis using clinicopathological features (tumor grade and cancer stage), we found that* VCAN*, *EFNA4*, and *LUM* have significantly higher expressions in patients with tumor grade than in healthy individuals (Figure 8A-C). Additionally, the expression of *VCAN*, *EFNA4*, and *LUM* was analyzed in each GC stage (I, II, III, and IV). *EFNA4* was highly overexpressed in all GC stages, whereas *VCAN* and *LUM* were highly overexpressed in GC stages II, III, and IV (Figure 8A-C). Therefore, LUM, EFNA4, and VCAN are considered useful prognostic indicators.

### Construction of prognostic model with three prognostic genes

**Figure 9:**
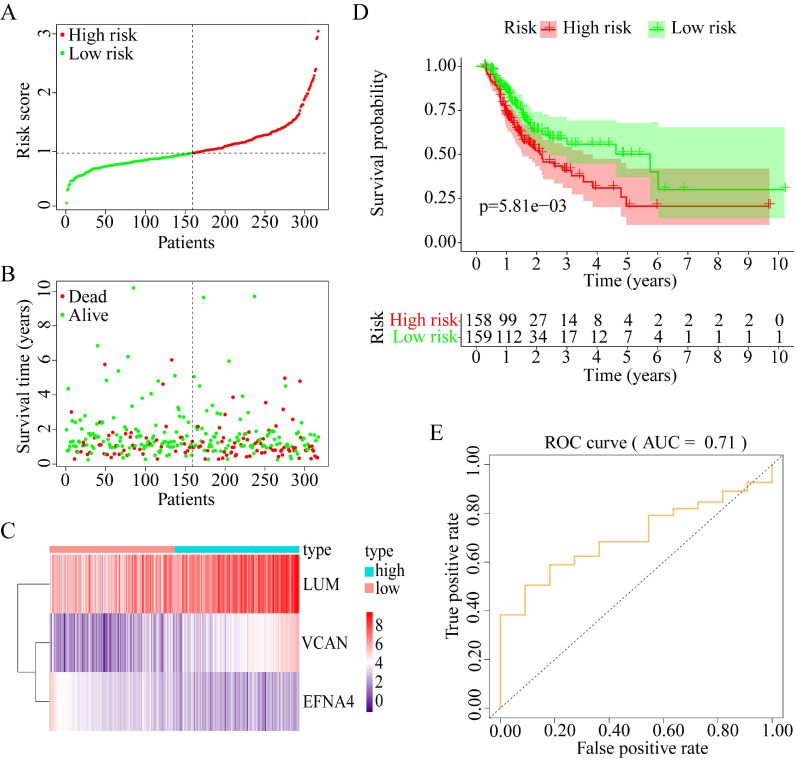
Construction of progonostic model with 3 prognostic genes identified. (A) The risk score distribution. (B) The patients' survival status distribution. (C) The heat map of the 3 genes for low- and high-risk groups. In the heat map, each column represents one sample, and each row represents one gene, and the color gradient ranging from purple to red represents the changing process from down- to upregulation. (D) The Kaplan-Meier curves for low- and high-risk groups. (E) The ROC curves for predicting OS in GC patients by the risk score.

To explore whether the combination of these three genes performed well in survival prediction, the prognostic model was constructed with three prognostic genes. Figure 9A-C shows the prognostic risk score results. OS was significantly different between the high-risk and low-risk groups (P < 0.01, Figure 9D). The 5-year OS was 50.21% (95% CI = 37.17-67.82%) in the low-risk group and 20.70% (95% CI = 10.17-42.05%) in the high-risk group. Besides, the AUC was 0.71 for 5-year OS, which implied that the prognostic model constructed with the three prognostic genes had a good accuracy for survival prediction (Figure 9E).

### Verification of the expression of the prognostic factors

**Figure 10:**
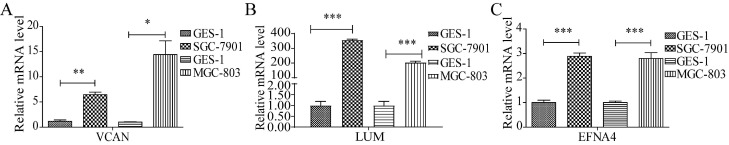
The expression of useful prognostic indicators in cell lines. *P<0.05; **P<0.01; ***P<0.001.

To verify the expression of *LUM*, *VCAN*, and *EFNA4*, real-time quantitative PCR analysis of GES-1 and SGC-7901 and MGC-803 cells was performed. The results revealed that the expression of *VCAN*, *LUM*, and *EFNA4* were higher in SGC-7901 and MGC-803 cells than in GES-1 cells, as shown in Figure 10. In addition, the expression of the 3 prognostic genes was also verified using GSE56807 dataset and the integrated dataset from UCSC Xena database, as shown in Figure S4. The results displayed that the 3 prognostic genes were significantly upregulated in GC samples.

### OS analysis of the prognostic indicator gene isoforms

**Figure 11:**
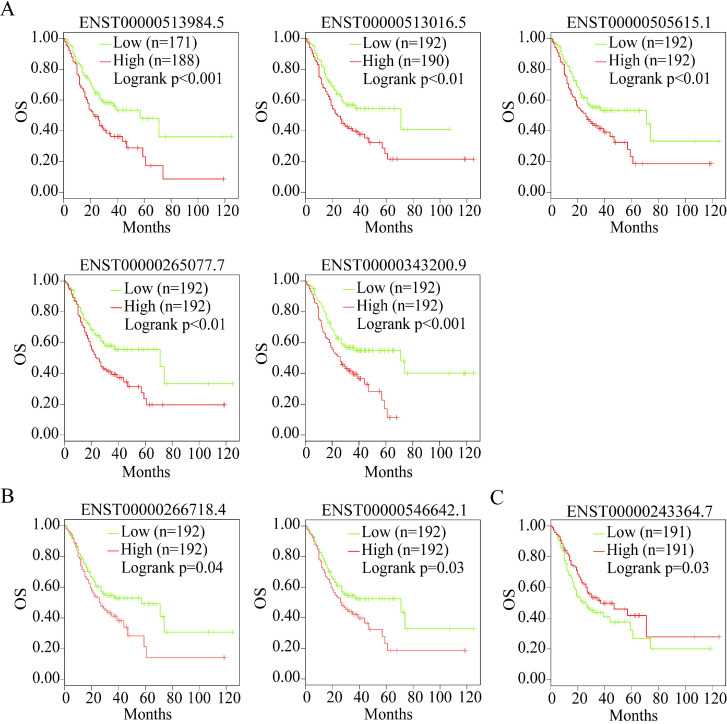
OS analysis of the capable prognostic gene isoforms. (A) *VCAN*. (B) *LUM*. (C) *EFNA4.*

Alternative splicing results in different transcripts; these transcripts are translated to different proteins and perform different biological functions. Therefore, we analyzed the isoforms of the three useful prognostic indicators of GC. Their isoforms were obtained via GEPIA2 analysis (Table 2). All transcripts of the three useful prognostic indicators were selected to explore their OS using GEPIA2 analysis. Two isoforms of *LUM*, five of *VCAN*, and one of *EFNA4* showed significant differences in terms of OS (*P* < 0.05) (Figure 11).

Therefore, the transcripts of the three useful prognostic indicators (*VCAN*, *LUM*, and* EFNA4*) that showed a difference in OS were defined as the valid prognostic indicators of GC (Table 2, bold).

### Correlation analysis between the useful prognostic indicators and tumor-infiltrating immune cells

The tumor microenvironment mainly contains tumor-infiltrating immune cells. Several studies have documented the presence of an association between tumor-infiltrating immune cell levels and tumor cell proliferation and metastasis, therapeutic response, and prognosis 21-23. They are thought to have a critical relationship with therapeutic response and prognosis 24. Therefore, the correlation of the useful prognostic indicators verified above with six types of tumor-infiltrating immune cells (B cells, CD4^+^ T cells, CD8^+^ T cells, neutrophils, macrophages, and dendritic cells) was investigated using TIMER.

**Figure 12:**
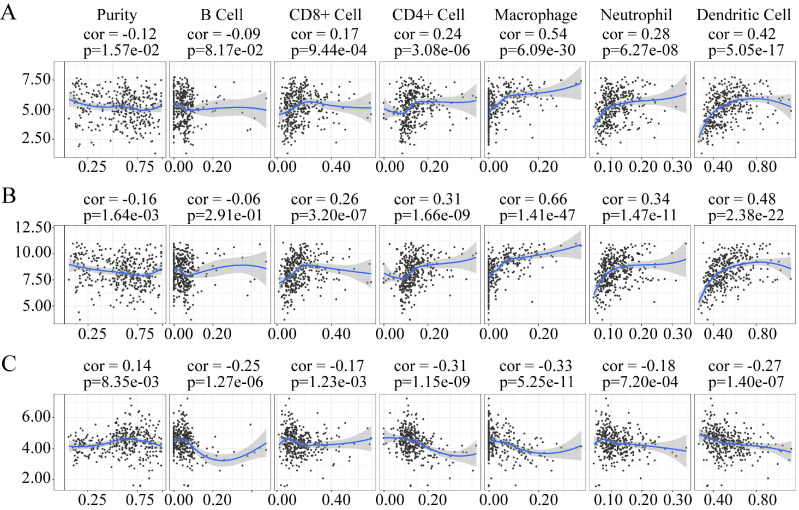
Association of *VCAN* (A), *LUM* (B), *EFNA4* (C) with tumor-infiltration immune cells in GC. *P* <0 05 denotes significance. Each dot represents a sample in the TCGA-STAD dataset.

The expression of *LUM* and* VCAN* negatively correlated with tumor purity, whereas the expression of *EFNA4* positively correlation with tumor purity (Figure 12). The expression of *LUM*, and *VCAN* had a noticeable positive correlation with infiltrating levels of CD8^+^ T cells, CD4^+^ T cells, macrophages, neutrophils, and dendritic cells in GC but had no apparent correlation with B cells (Figure 12A-B). A negative correlation was observed between the expression of *EFNA4* and the six types of tumor-infiltrating immune cells (Figure 12C).

## Discussion

Using integrative bioinformatics, 10 hub genes were identified in the PPI network of GC and their upregulation was validated in GC tissues and cell lines. Most of these genes closely correlated with the cell cycle. As hub genes, these genes may also have the potential to act as diagnostic genes. It has been reported that *CCNB1* 25, *CDK1* 26, *MAD2L1* 27, *CDC20* 28, and *CDC6* 29 could be essential genes for GC diagnosis and that they directly or indirectly contribute toward cell proliferation and metastasis as well as toward other biological functions associated with the cell cycle. The regulatory role of *CCNA2* in the MET-mediated cell-cycle pathway is reportedly blocked by miR-381-3p, which promotes the proliferation and metastasis of bladder cancer cells 30. *BUB1B* can also be activated by Forkhead box protein M1 to promote cell proliferation in glioblastoma 31. Nevertheless, studies on the regulatory mechanisms of *CCNA2* and *BUB1B* in GC are scarce. Regarding the other three genes, the upregulation of *TOP2A* enhanced the recurrence risk in patients with stage II/III GC, whereas its downregulation may play a significant role in chromosome instability and tumorigenesis. The expression of *TOP2A* is commonly altered at both the gene copy number and gene expression levels in cancer cells 32. *KIF11* is overexpressed in GC 33; its knockdown via RNAi inhibits the number and size of spheres formed in gastric cancer stem cells 34. AURKA activates HDM2, leading to the ubiquitination of p53; its inhibition markedly decreases cell survival *in vitro*, and *in vivo* in a xenograft tumor growth model, suggesting that *AURKA* expression can indicate a poor response to chemotherapy in GCs 35. All these genes appear to be promising candidate markers for GC therapy or diagnosis.

The three prognostic genes (*LUM*,* VCAN*, and *EFNA4*), with an obvious difference in OS and DFS, were highly expressed in patients with II, III, and IV GC. *VCAN* is highly expressed in advanced-stage GC and *VCAN* contributes to cell proliferation, cell differentiation, and tumor growth in GC 36. Interestingly, *VCAN* can control tumor metastasis and may identify previously undetected therapeutic targets to treat metastatic diseases in patients with breast cancer 37. The expression of *LUM* is closely associated with organ metastasis, lymphatic metastasis, and histological type in GC 38, 39.* EFNA4* encodes the human protein ephrin-A4, a member of the ephrin family 40. Ephrins are the ligands to Eph receptors and stimulate bi-directional signaling of the Eph/ephrin axis 41. Eph receptor and ephrin overexpression can result in tumorigenesis as related to tumor growth and survival and is associated with angiogenesis and metastasis in many types of human cancer 42. *EFNA4* upregulation in GC tissues has been identified using integrated transcriptomic and computational analysis methods 43. Ephrin-A4 binds to and phosphorylate the receptor Eph A2-8 and is significantly overexpressed in liver cancer and glioblastoma 42. In addition, the elevated expression of Eph-A4 in non-small cell lung carcinoma patients is also found to be significantly associated with favourable prognosis 44, which is similar to our finding about ephrin-A4. However, the detail mechanism of *EFNA4* influencing the tumorgenesis, development and prognostic of GC needs further study. Our finding about *LUM*,* VCAN*, and *EFNA4* that are highly expressed in GC cells proves they could be useful prognostic indicators of GC.

The effectiveness of the isoforms of *LUM*,* VCAN*, and *EFNA4* as the prognostic indicators of GC was also confirmed by OS analysis. However, to the best of our knowledge, the feasibility of the isoforms of *LUM*,* VCAN*, and *EFNA4* as prognostic indicators of GC has not been reported to date. Further studies are needed to verify these findings.

Because tumor-infiltrating immune cells have a clear relationship with tumor diagnostic and prognostic assessment 45, we explored the correlation between the three useful prognostic indicators and six types of tumor-infiltrating immune cells using TIMER. *LUM* and *VCAN* positively correlated with CD8^+^ T cells, CD4^+^ T cells, macrophages, neutrophils, and dendritic cells. In contrast,* EFNA4* negatively correlated with the six types of tumor-infiltrating immune cells. *LUM* and* VCAN* are mainly expressed on both T cells (CD8^+^ T cells and CD4^+^ T cells) and antigen-presenting cells (macrophages, neutrophils, and dendritic cells). *EFNA4* is not expressed on immune cells but is expressed on tumor cells. Therefore, all those three useful prognostic indicators, *LUM*,* VCAN*, and *EFNA4* are considered to have a relationship with the immunoregulation of the tumor environment.

## Conclusion

By analyzing the GC data from TCGA and two microarrays with combined bioinformatics tools, 10 hub genes and 3 useful prognostic indicators were identified as the possible indicators for future GC diagnosis and treatment. Identification of the correlation between the prognostic indicators and tumor-infiltrating immune cell levels in GC showed that three prognostic indicators play a role in cancer immunoregulation, which may be useful in cancer immunotherapy.

## Supplementary Material

Supplementary figures and tables.

## Figures and Tables

**Table 1:** Prognostic value of the 13 genes in the GC patients of the TCGA cohort

Gene symbol	Univariate analysis		Multivariate analysis		
	HR (95% CI)	P-value	HR (95% CI)	P-value	Coefficient
LINC01094	1.771 (1.322-2.372)	<0.001	1.803 (1.247-2.606)	0.002	0.589
CKMT2	1.118 (1.029-1.216)	0.009	1.152 (1.041-1.275)	0.006	0.141
LUM	1.001 (1.000-1.002)	0.020	0.997 (0.995-1.000)	0.022	-0.003
PSCA	1.001 (1.000-1.001)	0.002	1.001 (1.000-1.001)	0.093	0.001
TFF1	1.000 (1.000-1.000)	0.003	1.000 (1.000-1.000)	0.077	0.0002
FAP	1.071 (1.006-1.142)	0.033	0.835 (0.697-0.999)	0.048	-0.181
VCAN	1.022 (1.008-1.036)	0.002	1.027 (1.001-1.053)	0.044	0.026
FEN1	0.968 (0.943-0.994)	0.015	0.969 (0.941-0.997)	0.032	-0.032
CTHRC1	1.011 (1.004-1.018)	0.001	1.016 (1.003-1.028)	0.012	0.016
CDC6	1.004 (1.001-1.007)	0.015	1.007 (1.004-1.011)	0.000	0.007
PRRX1	1.052 (1.010-1.096)	0.015	1.083 (0.979-1.197)	0.120	0.080
EFNA4	0.965 (0.935-0.997)	0.031	0.970 (0.935-1.005)	0.096	-0.031
PMEPA1	1.003 (1.000-1.006)	0.037	1.003 (1.000-1.006)	0.070	0.003

**Table 2:** The isoforms of three prognostic genes

LUM_isoform	EFNA4_isoform	VCAN_isoform
ENST00000548071.1	ENST00000427683.2	ENST00000515397.1
**ENST00000546642.1**	ENST00000368409.7	**ENST00000513984.5**
**ENST00000266718.4**	ENST00000359751.8	ENST00000513960.5
	**ENST00000243364.7**	**ENST00000513016.5**
		ENST00000512590.6
		ENST00000507162.1
		**ENST00000505615.1**
		ENST00000503923.1
		ENST00000502527.2
		**ENST00000343200.9**
		ENST00000342785.8
		**ENST00000265077.7**

## Abbreviations

GC: gastric cancer
OS: overall survival
GEO: Gene Expression Omnibus
RNA-seq: RNA-sequencing
DEGs: differentially expressed genes
STAD: stomach adenocarcinoma
TCGA: The Cancer Genome Atlas
DFS: disease-free survival
BH: Benjamini & Hochberg
GO: Gene Ontology
KEGG: Kyoto Encyclopedia of Genes and Genomes
PPI: protein-protein interaction
CI: confidence interval
GEPIA2: Gene Expression Profiling Interactive Analysis
TIMER: Tumor IMmune Estimation Resource
