# Supplementary material for: Identification of the hub genes and prognostic indicators of gastric cancer and correlation of indicators with tumor-infiltrating immune cell levels
Source: J Cancer. 2021 May 13;12(13):4025–38. doi: 10.7150/jca.52105 (PMC8176244; doi:10.7150/jca.52105)
Supplement: Supplementary file 1 — Supplementary figures and tables. [file jcav12p4025s1.pdf]

Analysis Type by Cancer	Cancer vs. Normal CCNB1		Cancer vs. Normal CDK1		Cancer vs. Normal MAD2L1		Cancer vs. Normal AURKA		Cancer vs. Normal BUB1B		Cancer vs. Normal CCNA2		Cancer vs. Normal CDC6		Cancer vs. Normal TOP2A		Cancer vs. Normal CDC20		Cancer vs. Normal KIF11	
Bladder Cancer	5		6		2		5		5		3		3		8		6		2	
Brain and CNS Cancer	4		9	1	6	1	3	1	5	3	4	1	3	1	12		5		9	
Breast Cancer	14		25	1	20	1	21	1	19		13	2	8	1	28	1	19		16	1
Cervical Cancer	3		3		4		3		3		2		2		4		3		3	
Colorectal Cancer	12		13		12		16		8		6		16		19		6		7	
Esophageal Cancer	2		3		3		3		3		1		1		2		2		1	
Gastric Cancer	4		6		7		4		2		3		2		10		2			
Head and Neck Cancer	8		7		10		10		4		4		5		10		8		6	
Kidney Cancer	3		2	1	6			2							3					
Leukemia	2	9	1	8	1	2	1	7		3	1	5	2	3	1	5	1	5	3	3
Liver Cancer	3		4		4		4		4		4		1		4		3		3	
Lung Cancer	12		16		15		14		15		13		9		21		12		13	
Lymphoma	8	1	6	1	5		5	1	3	1	3	1	5		9		8	1	4	
Melanoma	2		1		2				1	1					3	1	1			
Myeloma		1		2								1						2		
Other Cancer	5	1	7	3	5	1	3	1	6	2	5		5		11	1	5	2	7	1
Ovarian Cancer	7		5		2		3		4		2		1		6		4		3	
Pancreatic Cancer	2		3		1		1	1	2	1	1		1		5		2		2	
Prostate Cancer	2						1				1				3					1
Sarcoma	9		12		12		9	1	12		12		5		12		12	1	11	
Significant Unique Analyses	105	12	128	17	116	5	105	15	95	11	77	10	68	5	169	8	97	11	88	6
Total Unique Analyses	351		361		363		340		325		373		358		368		321		336	

1 5 10 10 5 1
%

Figure S1. The expression of the 10 hub genes in GC (ONCOMINE). The numbers of datasets with 13 statistically significant mRNA upregulation (red) or downregulation (blue) of the 10 hub genes are shown. The upper label of the horizontal axis indicates the name of the hub genes; the lower label of the horizontal axis indicates the total number of datasets and the number of datasets with |FC| > 2 and P-value < 0.05.

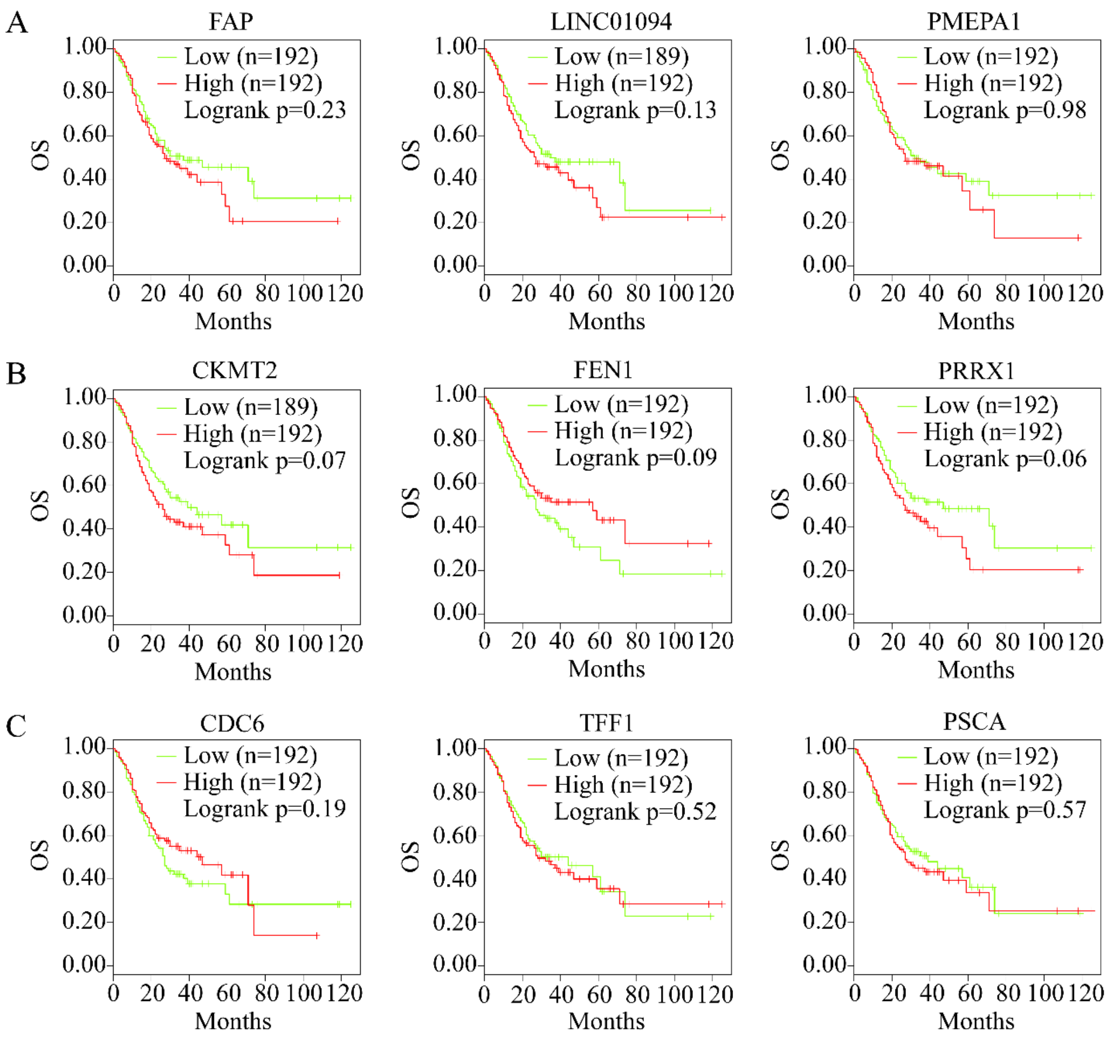

Figure S2. OS analysis of FAP, LINC01094, PMEPA1, CKMT2, FEN1, PRRX1, CDC6, TFF1, PSCA.

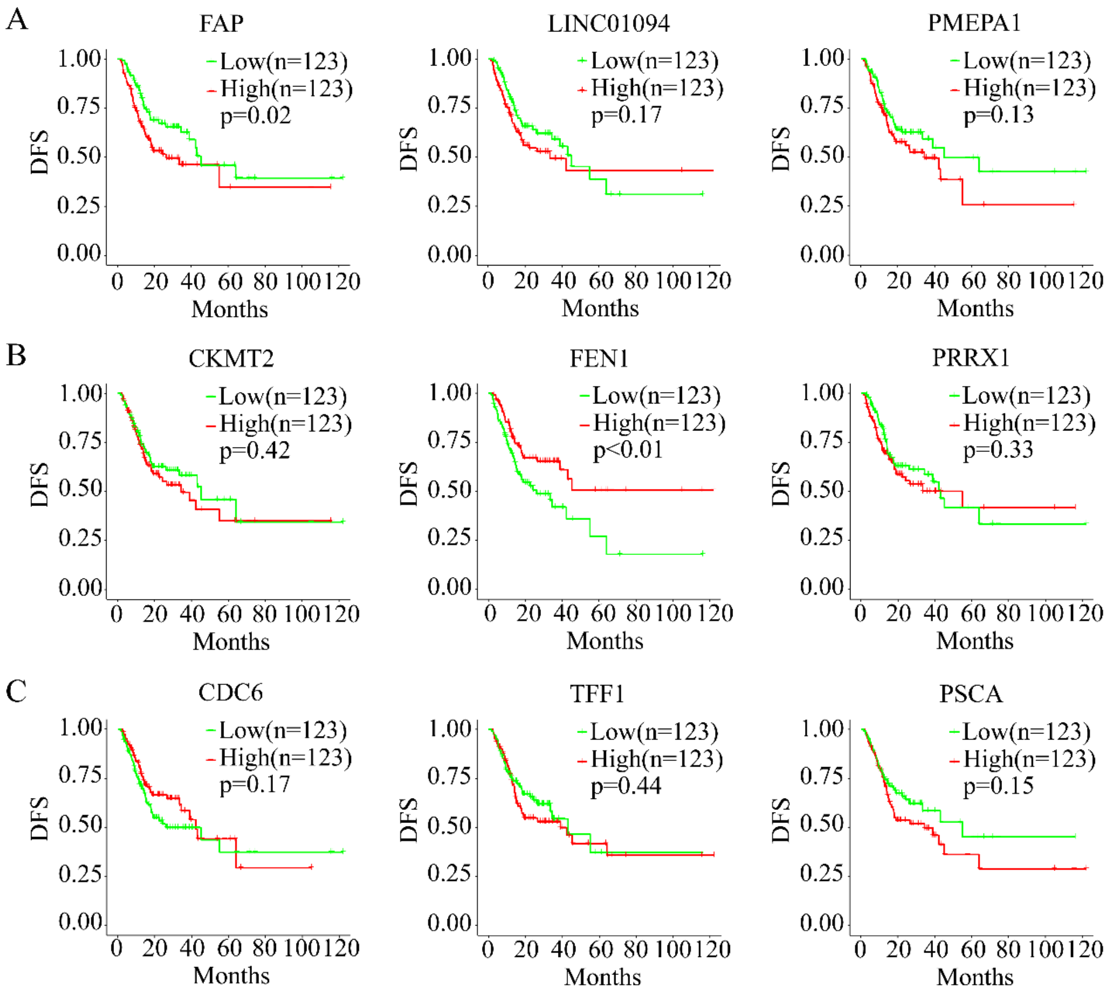

Figure S3. DFS analysis of FAP, LINC01094, PMEPA1, CKMT2, FEN1, PRRX1, CDC6, TFF1, PSCA.

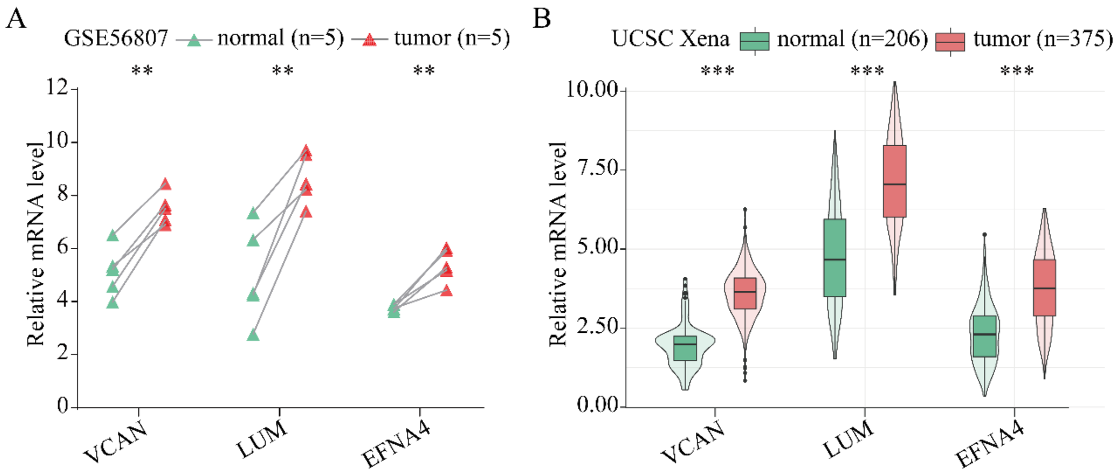

Figure S4. The expression of the three genes in GC and normal gastric tissues datasets. (A) The expression of the three genes in GSE56807 dataset with paired 5 GC and 5 normal gastric tissues samples. (B) The expression of the three genes in the integrated 206 normal gastric tissues samples and 375 GC tissues samples from UCSC Xena database. Expression values of genes are log2-transformed. **P* < 0.05; ***P* < 0.01; ****P* < 0.001.

Table S1 | Sequence Information

Name	Sequence
LUM-F	CTCGGCTTTGAGTTCTTACC
LUM-R	CTTGCAGCAGACAGACACC
VCAN-F	ATTACGGGTGGCTGTCGG
VCAN-R	TCTGCTATCAGGGGGAGGG
EFNA4-F	CTCGGCTTTGAGTTCTTACC
EFNA4-R	CTTGCAGCAGACAGACACC
GAPDH-F	GAACGGGAAGCTCACTGG
GAPDH-R	GCCTGCTTCACCACCTTCT
CDC6-F	CTGTTACCATCAACTTCTGAG
CDC6-R	CTGTTACCATCAACTTCTGAG
CDC20-F	AATGTGTGGCCTAGTGCTCC
CDC20-R	AGCACACATTCCAGATGCGA
BUB1B-F	TGAGGCAGCAATCTGTGAG
BUB1B-R	GAATCCACGATCCCTATGA
TOP2A-F	CTAGTTAATGCTGCGGACAAC
TOP2A-R	CATTTCGACCACCTGTCACTT
CDK1-F	CGGTTGACATCTGGAGTATAGG
CDK1-R	GCCACACTTCGTTGTTAGGA
AURKA-F	AAAGAGCAAGCAGCCCCTGC
AURKA-R	GAATTCAACCCGTGATATTCTT
CCNA2-F	TGGAAAGCAAACAGTAAACAGCC
CCNA2-R	GGGCATCTTCACGCTCTATTT
CCNB1-F	CTTGCAGTAAATGATGTGGATG
CCNB1-R	GTGACTTCCCGACCCAGTAG
MAD2L1-F	GTTCTTCTCATTCGGCATCAACA
MAD2L1-R	GAGTCCGTATTTCTGCACTCG
KIF11-F	GAACAATCATTAGCAGCAGAA
KIF11-R	TCAGTATAGACACCACAGTTG

Table S2 | The mRNA levels of 10 hub genes in different types of GC tissues and normal tissues at transcriptome level (ONCOMINE)

Genes	Type	Fold change	P-value	t-Test	Preferences
CCNB1	Gastric Mixed Adenocarcinoma	2.41	1.12E-4	4.43	(1)
CDK1	Gastric Intestinal Type Adenocarcinoma	2.54	7.42E-13	9.47	(2)
MAD2L1	Gastric Mixed Adenocarcinoma vs. Normal	3.77	7.12E-6	6.39	(1)
	Gastric Intestinal Type Adenocarcinoma	2.59	3.28E-5	4.67	(3)
AURKA	Gastric Intestinal Type Adenocarcinoma	3.07	1.78E-5	4.86	(3)
BUB1B	Gastric Intestinal Type Adenocarcinoma	3.56	1.80E-11	8.31	(4)
CCNA2	Gastric Intestinal Type Adenocarcinoma	4.07	1.08E-5	5.06	(3)
	Gastric Mixed Adenocarcinoma	2.06	6.20E-4	3.85	(1)
CDC6	Gastric Intestinal Type Adenocarcinoma	6.92	2.79E-6	5.41	(3)
TOP2A	Gastric Intestinal Type Adenocarcinoma	5.65	3.19E-7	6.06	(3)
	Gastric Mixed Adenocarcinoma	3.90	3.08E-5	5.49	(4)
CDC20	Gastric Intestinal Type Adenocarcinoma	3.06	6.73E-5	4.58	(3)
KIF11	Gastric Intestinal Type Adenocarcinoma	2.04	0.02E-1	3.25	(3)

References:

1. Cho JY, Lim JY, Cheong JH, et al. Gene Expression Signature-Based Prognostic Risk Score in Gastric Cancer. Clin Cancer Res. 2011;17(7):1850-7.

2. Chen X, Leung SY, Yuen ST, et al. Variation in gene expression patterns in human gastric cancers. Mol Biol Cell. 2003;14(8):3208-15.

3. Förster S, Gretschel S, Jöns T, et al. THBS4, a novel stromal molecule of diffuse-type gastric adenocarcinomas, identified by transcriptome-wide expression profiling. Mod Pathol. 2011;24(10):1390-403.

4. D'Errico M, Rinaldis ED, Blasi MF, et al. Genome-wide expression profile of sporadic gastric cancers with microsatellite instability. Eur J Cancer. 2009;45(3):0-469.
